# Capability of Machine Learning Algorithms to Classify Safe and Unsafe Postures during Weight Lifting Tasks Using Inertial Sensors

**DOI:** 10.3390/diagnostics14060576

**Published:** 2024-03-08

**Authors:** Giuseppe Prisco, Maria Romano, Fabrizio Esposito, Mario Cesarelli, Antonella Santone, Leandro Donisi, Francesco Amato

**Affiliations:** 1 Department of Medicine and Health Sciences, University of Molise, 86100 Campobasso, Italy; g.prisco2@studenti.unimol.it (G.P.); antonella.santone@unimol.it (A.S.); 2 Department of Information Technology and Electrical Engineering, University of Naples Federico II, 80125 Naples, Italy; mariarom@unina.it (M.R.); framato@unina.it (F.A.); 3 Department of Advanced Medical and Surgical Sciences, University of Campania Luigi Vanvitelli, 80138 Naples, Italy; fabrizio.esposito@unicampania.it; 4 Department of Engineering, University of Sannio, 82100 Benevento, Italy; mcesarelli@unisannio.it

**Keywords:** occupational ergonomics, load lifting, safe/unsafe posture, wearable sensors, inertial signals, machine learning, work-related musculoskeletal disorders

## Abstract

Occupational ergonomics aims to optimize the work environment and to enhance both productivity and worker well-being. Work-related exposure assessment, such as lifting loads, is a crucial aspect of this discipline, as it involves the evaluation of physical stressors and their impact on workers’ health and safety, in order to prevent the development of musculoskeletal pathologies. In this study, we explore the feasibility of machine learning (ML) algorithms, fed with time- and frequency-domain features extracted from inertial signals (linear acceleration and angular velocity), to automatically and accurately discriminate safe and unsafe postures during weight lifting tasks. The signals were acquired by means of one inertial measurement unit (IMU) placed on the sternums of 15 subjects, and subsequently segmented to extract several time- and frequency-domain features. A supervised dataset, including the extracted features, was used to feed several ML models and to assess their prediction power. Interesting results in terms of evaluation metrics for a binary safe/unsafe posture classification were obtained with the logistic regression algorithm, which outperformed the others, with accuracy and area under the receiver operating characteristic curve values of up to 96% and 99%, respectively. This result indicates the feasibility of the proposed methodology—based on a single inertial sensor and artificial intelligence—to discriminate safe/unsafe postures associated with load lifting activities. Future investigation in a wider study population and using additional lifting scenarios could confirm the potentiality of the proposed methodology, supporting its applicability in the occupational ergonomics field.

## 1. Introduction

Work-related musculoskeletal disorders (WMSDs) represent a significant health concern that affects millions of workers worldwide. These disorders encompass a broad range of painful and debilitating conditions that impact musculoskeletal structures. The risk of developing WMSDs is primarily associated with occupational tasks, and it is often the result of biomechanical overload, as has been reported in several studies [1,2,3,4].

In the last years, the prevalence of jobs involving repetitive movements, heavy lifting, and awkward postures has substantially risen; indeed, intensity, repetition, and duration represent the three elements that have the most impact on biomechanical risk during manual tasks [5]. Therefore, several quantitative and semi-quantitative methods have been proposed and implemented in occupational ergonomics to assess the biomechanical risk exposure [6,7,8,9,10]. Currently, wearable sensors are spreading in the field of occupational ergonomics as a valid tool to integrate methodologies which have already been experimented with, revolutionizing the way we monitor work activities and assess biomechanical risk [11,12,13,14,15,16,17]. Among these devices, inertial wearable sensors, which allow for the acquisition of linear acceleration and angular velocity, as well as wearable sensors for surface electromyography (sEMG) and pressure insoles, have proven to be useful for monitoring workers’ activities and assessing biomechanical risk [18,19,20,21].

Additionally, the integration of wearable sensors and artificial intelligence (AI) algorithms is increasingly strengthening in the field of occupational ergonomics, as has been reported in several scientific works. For instance, Donisi et al. [22] proposed a methodology, based on machine learning (ML) models fed with time- and frequency- domain features extracted from inertial signals acquired from the sternum, to classify biomechanical risk during lifting tasks according to the Revised NIOSH (National Institute for Occupational Safety and Health) Lifting Equation. They employed a logistic regression (LR) model, reaching an accuracy classification equal to 82.8%. Conforti et al. [23] used a support vector machine (SVM) fed with time-domain features extracted from inertial signals to discriminate safe and unsafe postures, reaching an accuracy level equal to 99.4%, while Prisco et al. [24] studied the feasibility of several tree-based ML algorithms fed with time-domain features extracted from inertial signals using a single sensor placed on the sternum. Aiello et al. [25] analyzed the discrimination power of ML algorithms to classify low-duty and high-duty activities using information related to the exposure to vibration, which was captured by means of two accelerometers placed on the wrists; the k nearest neighbors (kNN) algorithm reached a classification accuracy equal to 94%. Zhao and Obonyo et al. [26] proposed a model for recognizing construction workers’ postures based on the combination of 5 IMUs and deep learning (DL) algorithms (i.e., convolutional long short-term memory). Antwi-Afari et al. [27] proposed a methodology to recognize workers’ activities associated with overexertion from data acquired by means of a wearable insole pressure system using ML and DL algorithms; they found that the best algorithm was random forest (RaF), with an accuracy of over 97%. Fridolfsson et al. [28] studied the feasibility of ML models, which were fed with features extracted from acceleration signals using a shoe-based sensors, to classify work-specific activities; RaF was the best algorithm, once again reaching an accuracy of up to 71%. Mudiyanselage et al. [29] analyzed the level detection of risk of harmful lifting activities characterized by the Revised NIOSH Lifting Equations using ML and DL algorithms fed with features extracted from thoracic and multifidus sEMG signals, while Donisi et al. [30] studied the feasibility of ML algorithms fed with frequency-domain features extracted from sEMG signals of erector spinae and multifidus muscles to discriminate the biomechanical risk associated with manual material liftings, highlighting that the best algorithm was SVM, with an accuracy equal to 96.1%.

Considering the increasing integration of wearable sensors and AI in the field of occupational ergonomics, the aim of this paper was to study the feasibility of several ML algorithms—fed with time- and frequency-domain features extracted from inertial signals (linear acceleration and angular velocity) acquired from a single inertial measurement unit (IMU) placed on the sternum—to classify safe and unsafe postures during load lifting tasks.

Thus, the proposed methodology could offer an improvement or a valid integration of the procedures already established in the occupational ergonomics field to recognize bad postures, limiting the potential biomechanical risk associated with them in workers.

Moreover, the use of a single sensor (placed on the sternum) and the type of sensor (inertial sensor) make this procedure applicable to the workplace, and not confined to the laboratory like the other methodologies proposed in the scientific literature that are based on optoelectronic systems.

## 2. Materials and Methods

### 2.1. The Mobility Lab System (APDM)

**Figure 1:**
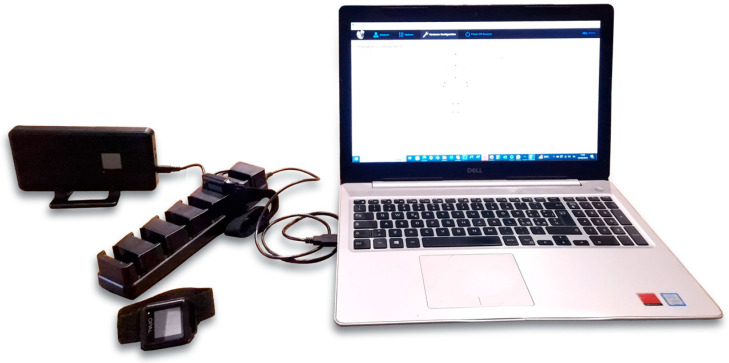
Mobility Lab System: access point, docking station, opal sensors, Mobility Lab software version 2.

**Figure 2:**
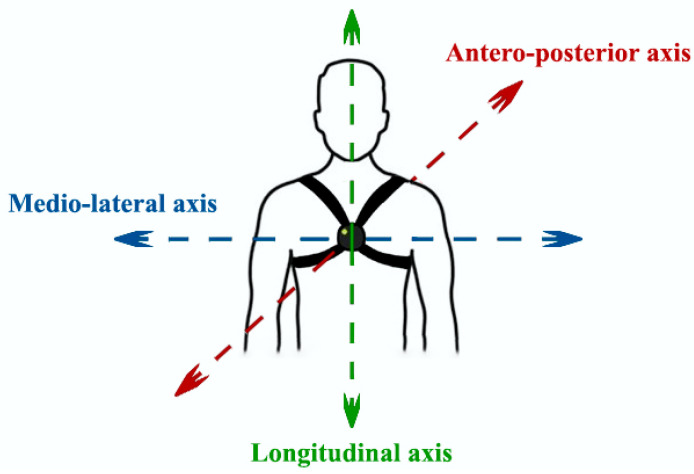
OPAL sensor placed on the sternum.

The Mobility Lab System (APDM wearable technologies Inc., Portland, OR, USA) is a technically advanced platform for the analysis of human movement, and is used in both clinical and research settings. In clinical practice, it is useful for treatment planning and monitoring patients. In research, it provides valuable data for scientific studies focused on mobility disorders [31,32,33]. This system is composed of both hardware and software components. The hardware is composed of an access point, a docking station, and inertial sensors (OPAL sensors), while the software is based on a dedicated application, namely, Mobility Lab software version 2 (Figure 1). The OPAL sensors are basically IMUs, which include tri-axial accelerometers (14-bit resolution, bandwidth of 50 Hz, and range of ±16 g), tri-axial gyroscopes (16-bit resolution, bandwidth of 50 Hz, and range of ±2000 deg/s), and tri-axial magnetometers (12-bit resolution, bandwidth of 32.5 Hz, and range of ±8 Gauss). These sensors allow for linear acceleration and angular velocity signals to be acquired with a sampling frequency up to 200 Hz. Moreover, the sensors are charged and configurated by means of the docking station. The access point provides the system with wireless communication capability by means of the Bluetooth 3.0 protocol, allowing for real-time data transmission from OPAL sensors to a host computer. Finally, the Mobility Lab software produces detailed reports based on objective metrics related to gait, balance, and movement patterns. In the present work, a single OPAL sensor placed on the sternum was used (Figure 2). The Mobility Lab System has been proven to be repeatable and accurate [34,35].

### 2.2. Study Population

In this study, 15 healthy subjects—9 men and 6 women—between the ages of 22 and 55 years old were enrolled. The subjects were selected excluding those who were affected by musculoskeletal disorders or other occupational pathologies. The anthropometric characteristics of the study population are shown in the Table 1.

### 2.3. Experimental Study Protocol

**Figure 3:**
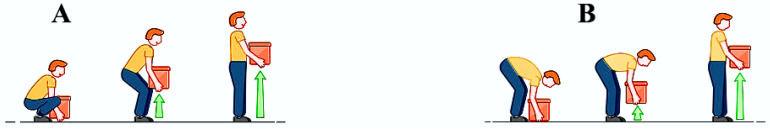
Phases of load lifting execution associated with safe posture (**A**) and unsafe posture (**B**).

Each subject participated in a session divided into two trials. The first trial consisted of 20 consecutive liftings according to the squat technique—namely, with back extended, legs flexed, rigid arms, and trunk flexed at the hip joints—associated with a safe posture. The load had to be gripped while keeping the legs apart—with a distance between the feet of 20/30 cm—in order to ensure balance during lifting (Figure 3A). The second trial consisted of 20 consecutive liftings associated with an unsafe posture; the liftings were performed with a curved back and non-flexed legs (Figure 3B). Each lifting task was carried out using a plastic container (56 × 35 × 31 cm^3^) with weights equally distributed inside. Squat and stoop techniques were widely regarded as the “correct” and “incorrect” techniques for lifting activities, as has been reported in several articles in the scientific literature [36,37]. The details regarding the execution of the study protocol are reported in the Table 2.

### 2.4. Digital Signal Processing and Feature Extraction

The linear acceleration and angular velocity signals were acquired for each subject during the lifting tasks. The inertial signals were appropriately segmented in order to extract the portion of the signals in the time windows corresponding to the lifting actions. All signals were segmented starting from the segmentation carried on the acceleration signal along the *x*-axis or longitudinal axis (see Figure 2). The choice of the signal to be segmented fell on the acceleration signal along the *x*-axis, since the acceleration component along the longitudinal axis—i.e., the axis relating to the lifting of the load—had a more enhanced waveform in terms of amplitude to encourage segmentation.

**Figure 4:**
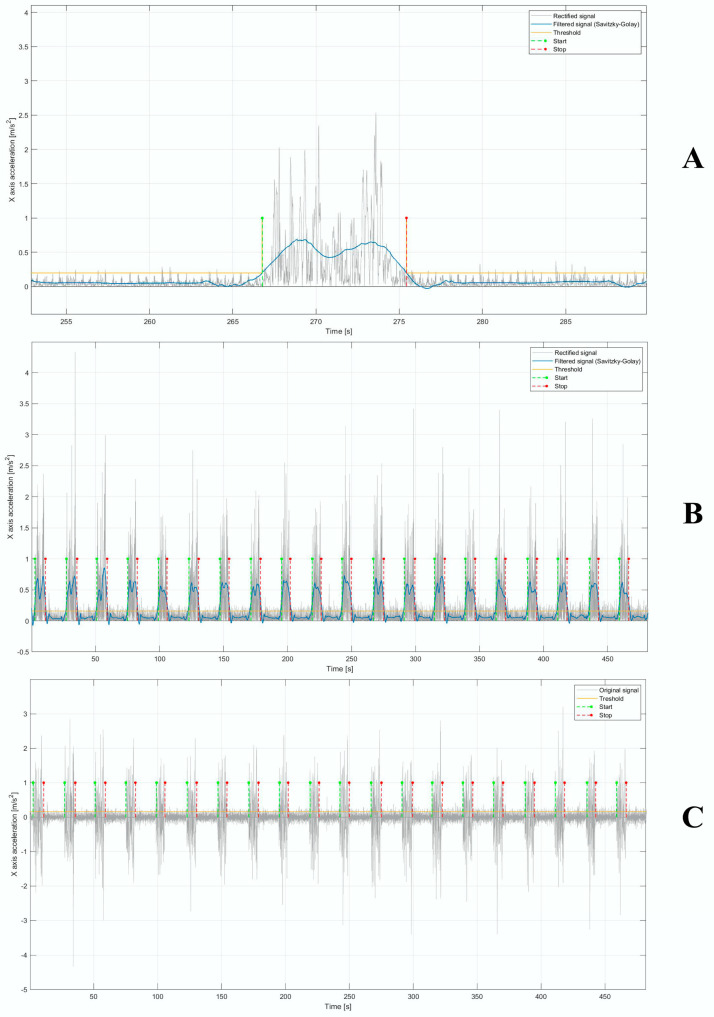
(**A**) Rectified original signal (in grey); rectified and filtered signal using Savitzky–Golay filter (in blue) and threshold (in yellow) to detect the start and stop points (in green and red, respectively). A single lifting is shown. (**B**) Rectified original signal (in grey); rectified and filtered signal using Savitzky–Golay filter (in blue) and threshold (in yellow) to determinate the start and stop points (in green and red, respectively). All the liftings of a single trial are shown. (**C**) Original acceleration signal and start and stop points detected to identify the ROIs.

We performed 3 steps to segment the signals. Firstly, the original signal was filtered using a 4° order Butterworth band-pass filter with a band pass ranging from 1 to 50 Hz in order to remove mainly the continuous or DC component. Secondly, the signal was rectified and then filtered by means of a Savitsky–Golay filter [38], with a polynomial order and frame length equal to 4 and 1101, respectively. Finally, an empirical threshold for each subject was set; therefore, from the intersection between the threshold and the final filtered signal, the start and stop points—necessary to segment the signal in the individual region of interest (ROI) corresponding to the lifting tasks—were detected (Figure 4A,B). From our knowledge of the start and stop points, we extracted the ROIs on the original signal (Figure 4C).

For each ROI, several time- and frequency-domain features were extracted. The following time-domain features were extracted:

- Standard deviation (STD) (m/s^2^ for acceleration, deg/s for angular velocity):

(1) STD=1N−1∑1N(xi−x¯)212

- Mean absolute value (MAV) (m/s^2^ for acceleration, deg/s for angular velocity):

(2) $$MAV=\frac{1}{N}\sum_{1}^{N}\left|x_{i}\right|$$

- Peak to peak amplitude (PP) (m/s^2^ for acceleration, deg/s for angular velocity):

(3) $$PP=\left|\max\left(x_{i}\right)-min(x_{i})\right|$$

- Zero crossing rate (ZCR) (adim):

(4) $$\left\{x_{i}<0\;and\;x_{i+1}>0\right\}\;or\;\left\{x_{i}>0\;and\;x_{i+1}<0\right\}$$

- Slope sign changes (SSC) (adim):

(5) $$\left\{x_{i}<x_{i+1}\;and\;x_{i}<x_{i-1}\right\}\;or\;\left\{x_{i}>x_{i+1}\;and\;x_{i}>x_{i-1}\right\}$$

Concerning frequency-domain features, the total power spectrum (TPS), computed using the fast Fourier transform (fft) algorithm, was considered to extract the related features. The following features were extracted:

- Total power (P) (m/s^2^ for acceleration, deg/s for angular velocity):

(6) $$P=\frac{1}{N}\sum_{1}^{N}{S_{x}}_{i}$$

(7) $${S_{x}}_{i}={\left|X_{i}\right|}^{2}$$

- Spectral entropy (SE) (adim):

(8) $$SE=-\sum_{1}^{N}p_{i}\cdot \;\log_{2}p_{i}$$

(9) $$p_{i}=\frac{{S_{x}}_{i}}{\sum_{j=1}^{N}{S_{x}}_{j}}$$

- Kurtosis (Kurt) (adim):

(10) $$Kurt=\frac{\sum {\left({S_{x}}_{i}-\bar{S_{x}}\right)}^{3}}{N\cdot s^{4}}-3$$

- Skewness (Skew) [adim]:

(11) $$Skew=\frac{\sum {\left({S_{x}}_{i}-\bar{S_{x}}\right)}^{3}}{N\cdot s^{3}}$$

where:

- $x_{i}$: *i*-th sample of the signal;
- N: number of samples of the signal;
- $X_{i}$: *i*-th sample of the Fourier transformation of the signal;
- ${S_{x}}_{i}$: *i*-th sample of the TPS of the signal;
- $\bar{S_{x}}$: mean of the TPS of the signal;
- s: STD of the TPS of the signal.

The aforementioned features were implemented according to the following references: absolute arithmetic mean [39], standard deviation [40], peak to peak amplitude [41], zero crossing rate [39], slope sign changes [39], total power [40], entropy [42], kurtosis [40], and skewness [40].

### 2.5. Statistical Analysis

A statistical analysis was carried out to verify which features presented a statistically significant difference in order to discriminate safe/unsafe postures during weight lifting. A Shapiro–Wilk normality test was performed to evaluate the normality of each feature in order to choose the correct parametric (*t*-test) or non-parametric (Wilcoxon test) two-tailed paired test. For all the statistical tests, a confidence level equal to 95% was chosen (definition of statistical significance: *p*-value < 0.05).

Statistical analysis was performed using JASP 0.17.1 (University of Amsterdam, Amsterdam, The Netherlands).

### 2.6. Machine Learning Analysis

ML is a field in which predictive models are created to learn or improve their performance based on input data observation [43]. In this study, the features extracted for each ROI of the inertial signals were used to build a supervised dataset to feed ML algorithms with the goal of performing a binary classification (safe and unsafe posture classification).

Supervised learning is an important branch of ML which creates models based on labeled data training [44]. In the present study, the following 8 supervised ML algorithms were implemented to assess their classification accuracy in order to discriminate safe and unsafe postures: support vector machine (SVM) [45]; decision tree (DT) [46]; gradient boosted tree (GB) [47]; random forest (RaF) [48]; logistic regression (LR) [49]; k nearest neighbor (kNN) [50]; multilayer perceptron (MLP) [51]; and probabilistic neural network (PNN) [52].

For all the ML algorithms, the hyperparameters’ optimization was performed to maximize the classification accuracy. Regarding SVM, a polynomial kernel with bias, power, and gamma equal to 1.141, 1.734, and 1.489 was set, respectively. For kNN, a k equal to 7 was set. Concerning LR, a step size, maximum of epoch, and epsilon equal to 0.516, 111, and 0.004 were chosen, respectively. Regarding DT, minimum number records per node were set equal to 5, number records to store per view were set equal to 6939, and the maximum nominal value was set equal to 4. Moreover, pruning was not implemented. Concerning GB, the maximum of levels was set equal to 2, the number of models was set equal to 149, and the learning rate was set equal to 0.333. For RaF, the maximum of levels, number of models, and minimum node size were set equal to 5, 52, and 4, respectively. For MLP, the maximum of iterations, hidden layer, and number of hidden layers were chosen to be equal to 60, 1, and 5, respectively. Finally, for PNN, the theta minus was set equal to 0.109, and the theta plus was set equal to 0.928.

Moreover, for the LR, kNN, and SVM algorithms, the min-max (MM) normalization was performed so that all feature values were squeezed (or stretched) within the range of [0, 1]. The MM normalization was set because some models are sensitive to the scale of input features, while other models, such as tree-based models, are less sensitive [53].

As validation strategy, the leave-one-subject-out cross-validation (CV) strategy was adopted. It used each individual subject as a test set and the remaining ones as a training set. In this study, 14 subjects were used to train and 1 subject was used to test the predictive models; this procedure was executed in an iterative way 15 times so as to test the ML models on each subject.

Accuracy, F-measure, specificity, sensitivity, precision, recall, and area under the receiver operating characteristic curve (AUCROC) were used as evaluation metrics to assess the classification power of the proposed ML algorithms fed with the extracted features.

Moreover, a feature importance according to information gain (IG) method was computed. The IG approach—based on entropy—is an indicator of the importance of each feature to the target class [54].

The ML analysis was performed using the Knime Analytics Platform (version 4.1.3), a platform widely used in the biomedical engineering field [55,56,57].

## 3. Results

Firstly, a statistical analysis based on two-tailed paired tests—the parametric test (*t*-test) for features with a normal distribution and the non-parametric test (Wilcoxon test) for features with non-normal distribution—was performed to evaluate which features were statistically significant in order to discriminate the two target classes, namely, safe and unsafe postures. This analysis was carried out separately for acceleration and angular velocity, considering all axes (x, y, z). In Table 3 and Table 4, the results of the statistical analysis for linear acceleration and angular velocity, respectively, are shown.

Secondly, the feasibility of the eight ML algorithms—fed with time- and frequency-domain features extracted from inertial signals acquired by means of a single IMU placed on the sternum—to classify safe and unsafe postures was assessed. The supervised dataset consisted of 600 instances (15 subjects × 40 lifting instances), 54 features (9 features extracted × 2 signal (acceleration and angular velocity) × 3 axis (x, y, z) × 1 body position (sternum)), and 2 classes (safe posture, unsafe posture). The evaluation metric scores reached by the ML classifiers using the leave-one-subject-out CV strategy and hypermeters optimization are reported in Table 5.

**Figure 5:**
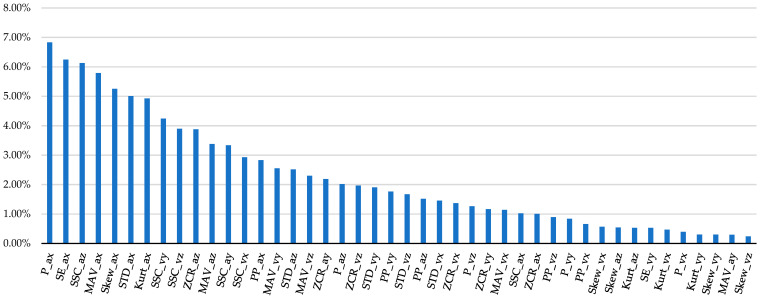
Ranking of the features extracted from acceleration and angular velocity signals along x, y, and z-axes (ax, ay, az, vx, vy, vz) according to the IG method. Spectral entropy (SE); kurtosis (Kurt); skewness (Skew); power (P); peak to peak amplitude (PP); standard deviation (STD); mean absolute value (MAV); zero crossing rate (ZCR); slope sign changes (SSC).

Finally, the feature importance—according to the IG method—is shown in Figure 5. The only features with non-zero ranking values are reported.

## 4. Discussion

The purpose of this work was to study the feasibility of ML models fed with time- and frequency-domain features extracted from inertial signals acquired by a single IMU placed on the sternum in order to automatically discriminate safe and unsafe posture during weight liftings.

Table 3 and Table 4 report the statistical analysis results based on two-tailed paired tests, and it is highlighted that almost all the features, for both acceleration and angular velocity, showed statistically significant differences discriminating safe and unsafe postures. The results showed that all features extracted from the angular velocity signal exhibited statistically significant differences between the two classes (*p* value lower than 0.001), while for the acceleration signal, PP_acc and MAV_acc did not exhibit statistically significant differences, with *p*-values equal to 0.105 and 0.127, respectively. This result suggests that the angular velocity has a discriminant power slightly higher than the acceleration signal in this context.

Considering the correlation existing among the instances (liftings) of the same subject, and to avoid training the algorithms on instances related to all the subjects, a ML analysis using the leave-one-subject-out CV strategy was performed in order to obtain more robust results. Table 5 shows the evaluation metric scores for each ML model fed with specific time- and frequency-domain features to classify safe and unsafe postures. Almost all of the ML algorithms reached a classification accuracy of more than 0.9, except for PNN and DT (accuracy equal to 0.88 ± 0.17 and 0.79 ± 0.16, respectively). The low accuracy value of the PNN algorithm could be due to the correlation existing between the features, since probabilistic classifiers make a basic assumption of independence among features, which is not always verified. The best ML algorithm was LR, with accuracy, F-measure, specificity, sensitivity, precision, recall, and AUCROC equal to 0.96 ± 0.11, 0.97 ± 0.08, 0.92 ± 0.21, 0.99 ± 0.01, 0.95 ± 0.12, 0.99 ± 0.01, and 0.99 ± 0.01, respectively. AUCROC values provide information about the ability of ML algorithms to discriminate between classes. Conventionally, AUCROC values are divided into three ranges: moderate discrimination power (values between of 0.70–0.80), good discrimination power (values between of 0.80–0.90), and excellent discrimination power (values greater than 0.90). Regarding LR, the AUCROC value was equal to 0.99 ± 0.01, demonstrating its excellent ability to discriminate safe and unsafe classes.

Figure 5 shows the feature importance results based on the IG method, and it emerged that 44 features out of 54 (81.5%) had non-zero values.

Considering all inertial signals, it emerged that the x, y, and z axes showed ranking values equal to 47.87%, 19.40%, and 32.72%, respectively. Therefore, the *x*-axis (i.e., vertical axis) was more representative than the y and z axes (i.e., medio-lateral and antero-posterior axes, respectively) in classifying the target classes. We expected this result, since the weight lifting was carried out along the x axis, namely, the vertical direction.

Considering all axes (x, y, z) and inertial signals, we highlight that SSC, MAV, STD, ZCR, P, PP, Skew, SE, and Kurt presented the following ranking values, respectively: 21.55%, 15.44%, 12.55%, 11.56%, 11.33%, 7.66%, 6.90%, 6.77%, and 6.22%. This result suggests that the features extracted from the time-domain were more predictive than frequency-domain features in classifying safe and unsafe postures during load liftings, with a ranking value equal to 61.11%. The use of time-domain features alone is relevant, since it could reduce the computational effort by allowing for a real-time analysis. However, it needs to be understood how much it affects the predictive ability of the ML models.

Different studies presented in the scientific literature have attempted to classify safe and unsafe postures during lifting actions using ML algorithms. Hung et al. [58] used a DL model fed with kinematic features extracted from a three-dimensional motion tracking system to classify three posture classes (stoop, stand, squat), reaching a classification accuracy of up to 94%. The limitation of the methodology described above, as highlighted by the authors themselves, was that the effect of holding the load while performing a lifting task was not considered, which determined a change in the classification accuracy. Another limitation is the very small study population, since they considered only two subjects, and therefore, the results cannot be considered robust.

In the study of Greene et al. [59], the authors evaluated the feasibility of DT model using a depth camera located in front of the sagittal plane of the subject to extract kinematic features in order to classify three posture classes (stoop, stand, squat), reaching a classification accuracy of up to 100%. The same authors [59] developed a methodology to automatically classify lifting postures—stoop, squat, and stand—using features obtained by drawing a rectangular bounding box tightly around the body on the sagittal plane in video recordings. A classification CART algorithm was used in this work, reaching an accuracy of up to 100%.

Chae et al. [60] proposed a methodology based on ML algorithms (SVM and RaF) and DL algorithms (artificial neural network (ANN)) fed with kinetic and kinematic features obtained from six cameras and two ground reaction forces for the binary classification of stoop and squat postures, reaching an accuracy equal to 94%.

Conforti et al. [23] studied the feasibility of an SVM algorithm with linear, polynomial (quadratic and cubic), and Gaussian kernels fed with time-domain features—extracted from inertial signals using eight IMUs applied on the upper and lower body segments of the subject—to classify correct and incorrect postures during load lifting, obtaining a classification accuracy greater than 90%. The limitation, as reported by the same authors, was that the high number of wearable sensors used—eight IMUs—allowed it to confine the load lifting assessment to the laboratory. On the other hand, contrary to our study, the extraction of only time-domain features lessened the computational burden, potentially allowing for the analysis of posture in real time.

Furthermore, Ryu et al. [61] assessed the action recognition of masonry workers by means of three ML classification algorithms—SVM, kNN, and MLP—fed with time- and frequency-domain features extracted from acceleration signals acquired from the wrists, reaching a classification accuracy between 80% and 100%.

O’Reilly et al. [62] explored the feasibility of a back-propagation neural network classifier fed with time-domain features, which were extracted from inertial signals using one IMU applied on lumbar, to classify correct and incorrect postures during squat lifting. The classifier was trained and tested using leave-one-subject-out CV, obtaining an accuracy equal to 80.45%.

Finally, Youssef et al. [63] assessed the feasibility of the ANN algorithm fed with kinematic features obtained from nine IMUs and one camera for binary classification between good and bad squat postures. The classifier was trained and tested using 10-fold CV, reaching an accuracy equal to 96%.

On the basis of the highlighted results and considering the number and the type of wearable sensors used in the previously proposed methodologies, it is possible to state that the proposed approach could solve the problem of poor applicability in the workplace by using a methodology based on a single inertial sensor placed on the sternum. Moreover, the results demonstrated the good discrimination power of the proposed methodology, as reported in the Table 5, although we used a simple technology (IMU) and a simple configuration (a single inertial sensor placed on the sternum) that make the procedure applicable in real-world settings (e.g., the workplace). Finally, the proposed methodology, from a cost-effectiveness point of view, is more economical compared to the other technologies proposed in the scientific literature, since it is based on accelerometers and gyroscopes that are cheaper if compared to cameras or optoelectronic systems.

Therefore, the proposed methodology—which combines ML algorithms and time- and frequency-domain features extracted from inertial signals acquired from a single IMU placed on the sternum—proved to be able to discriminate safe and unsafe postures during weight lifting.

## 5. Conclusions

The combination of specific features extracted from inertial signals acquired by means of a single IMU placed on the sternum and an ML algorithms allowed us to distinguish safe and unsafe postures. Interesting results were obtained, in particular, LR classifier reached high scores in evaluation metrics. The proposed methodology was able to discriminate safe and unsafe postures, making the procedure of posture assessment automatic, economic, non-time consuming, non-invasive, and not operator-dependent. Therefore, the proposed methodology could be of direct practical relevance for occupational ergonomics. Moreover, the use of a single IMU sensor allows this procedure to be applicable in the workplace, and not confined to the laboratory like the other methodologies proposed in the scientific literature that are based on optoelectronic systems. Although the use of several type of sensors can provide further information—i.e., the contribution of lower limb or trunk kinematics—the use of a single type of sensor, as in the case in this study, can make the procedure simpler and more applicable in the workplace. To reduce biomechanical overload and to prevent the occurrence of WMSD, the design of a real-time posture-monitoring platform based on our methodology could be a powerful solution in the workplace, although the analysis in the frequency domain could be limiting.

However, there are some limitations in this study that make the work preliminary. Firstly, only 15 subjects were enrolled in this experiment, and, secondly, we did not consider older people with comorbidities and/or bone fragility, conditions that can trigger or worsen WMSDs, affecting the accuracy of ML classification and participants’ health status if not conducted under medical supervision and with previously determined bone mineral, cardiorespiratory, postural, and fitness statuses. Future investigation in a large study population—in terms of samples and age—could confirm the potential of this methodology to classify safe and unsafe postures during weight lifting in order to offer a valid integration of the procedures already established in the occupational ergonomic field. As a future development, DL algorithms could be also investigated in order to explore their feasibility in discriminating safe and unsafe postures using the same dataset, as well as to understand if there would be improvements in terms of evaluation metrics. Moreover, it could be interesting to validate the proposed methodology by comparing it with methodologies already established in occupational ergonomics (e.g., PoseNet [64]).

Finally, it would be interesting to explore the long-term effectiveness and reliability of using machine learning algorithms and IMUs for ergonomic assessment, even if it is premature, considering the recent interest in the application of wearable technologies coupled with artificial intelligence in the occupational ergonomics field.

## Figures and Tables

**Table 1:** Anthropomorphic characteristics of the study population, reported as mean ± standard deviation.

Characteristics	
Age (years)	33.2 ± 7.8
Height (cm)	171.1 ± 8.3
Weight (kg)	66.1 ± 9.9
Body mass index (kg/m^2^)	22.5 ± 2.9

**Table 2:** Combination of weight, frequency, duration, and vertical displacement variables for lifting activities corresponding to safe and unsafe postures.

Parameters				
Vertical Displacement (Start–End) (cm)	Duration (min)	Frequency (lifting/min)	Weight Lifted (kg)	
			M	F
50–125	8	2.5	7	5

**Table 3:** Paired test between safe and unsafe postures for each feature extracted from acceleration signal (acc).

Features *	Safe Posture Mean ± STD	Unsafe Posture Mean ± STD	*p*-Value
SE_acc	0.569 ± 0.038	0.604 ± 0.029	<0.001
Kurt_acc	111.448 ± 39.195	82.120 ± 39.200	<0.001
Skew_acc	8.829 ± 1.580	7.346 ± 1.762	<0.001
P_acc	150.342 ± 96.122	96.717 ± 48.214	<0.001
PP_acc	3.809 ± 1.716	3.620 ± 1.073	0.105
STD_acc	0.480 ± 0.109	0.450 ± 0.114	<0.001
MAV_acc	0.330 ± 0.060	0.322 ± 0.078	0.127
ZCR_acc	102.112 ± 17.360	86.028 ± 21.610	<0.001
SSC_acc	350.304 ± 45.250	271.169 ± 55.144	<0.001

Definition of statistical significance: *p*-value < 0.05. * Spectral entropy (SE); kurtosis (Kurt); skewness (Skew); power (P); peak to peak amplitude (PP); standard deviation (STD); mean absolute value (MAV); zero crossing rate (ZCR); slope sign changes (SSC).

**Table 4:** Paired test between safe and unsafe postures for each feature extracted from angular velocity signal (vel).

Features *	Safe Posture Mean ± STD	Unsafe Posture Mean ± STD	*p*-Value
SE_vel	0.547 ± 0.039	0.559 ± 0.034	<0.001
Kurt_vel	119.318 ± 38.884	98.303 ± 34.176	<0.001
Skew_vel	9.458 ± 1.597	8.472 ± 1.554	<0.001
P_vel	8.566 ± 17.438	10.898 ± 6.859	<0.001
PP_vel	0.866 ± 0.832	1.064 ± 0.368	<0.001
STD_vel	0.105 ± 0.046	0.137 ± 0.042	<0.001
MAV_vel	0.073 ± 0.025	0.100 ± 0.031	<0.001
ZCR_vel	79.261 ± 16.200	63.158 ± 17.668	<0.001
SSC_vel	295.074 ± 39.092	223.753 ± 46.063	<0.001

Definition of statistical significance: *p*-value < 0.05. * Spectral entropy (SE); kurtosis (Kurt); skewness (Skew); power (P); peak to peak amplitude (PP); standard deviation (STD); mean absolute value (MAV); zero crossing rate (ZCR); slope sign changes (SSC).

**Table 5:** Evaluation metric scores reported as mean ± standard deviation using features extracted from inertial signals, leave-one-subject-out CV strategy, and hyperparameter optimization for each classification algorithm.

	SVM	DT	GB	RaF	LR	kNN	MLP	PNN
Accuracy	0.94 ± 0.12	0.88 ± 0.17	0.94 ± 0.10	0.95 ± 0.09	0.96 ± 0.11	0.91 ± 0.12	0.92 ± 0.15	0.79 ± 0.16
F-measure	0.95 ± 0.09	0.89 ± 0.14	0.94 ± 0.11	0.94 ± 0.13	0.97 ± 0.08	0.92 ± 0.09	0.94 ± 0.11	0.84 ± 0.10
Specificity	0.89 ± 0.24	0.82 ± 0.29	0.94 ± 0.11	0.95 ± 0.11	0.92 ± 0.21	0.84 ± 0.23	0.83 ± 0.30	0.61 ± 0.30
Sensitivity	0.99 ± 0.01	0.94 ± 0.14	0.95 ± 0.11	0.95 ± 0.17	0.99 ± 0.01	0.98 ± 0.04	1.00 ± 0.00	0.98 ± 0.04
Precision	0.92 ± 0.14	0.88 ± 0.17	0.95 ± 0.10	0.96 ± 0.08	0.95 ± 0.12	0.88 ± 0.15	0.90 ± 0.17	0.74 ± 0.15
Recall	0.99 ± 0.01	0.94 ± 0.14	0.95 ± 0.11	0.95 ± 0.17	0.99 ± 0.01	0.98 ± 0.04	1.00 ± 0.00	0.98 ± 0.04
AUCROC	0.99 ± 0.02	0.86 ± 0.21	0.99 ± 0.03	0.99 ± 0.01	0.99 ± 0.01	0.96 ± 0.07	0.99 ± 0.04	0.87 ± 0.19

Support vector machine (SVM), decision tree (DT), gradient boosted tree (GB), random forest (RaF), logistic regression (LR), k nearest neighbor (kNN), multilayer perceptron (MLP), probabilistic neural network (PNN), area under the receiver operating characteristic curve (AUCROC).

## Data Availability

The datasets generated and analyzed in this study are not publicly available due to the privacy policy, but are available from the corresponding author upon reasonable request.
